# In Vitro Anthelmintic Activity of *Isatis tinctoria* Extracts against Ewes’ Gastrointestinal Nematodes (GINs), a Possible Application for Animal Welfare

**DOI:** 10.3390/vetsci9030129

**Published:** 2022-03-10

**Authors:** Monica Ragusa, Natalizia Miceli, Cristian Piras, Antonio Bosco, Fabio Castagna, Laura Rinaldi, Vincenzo Musella, Maria Fernanda Taviano, Domenico Britti

**Affiliations:** 1 Department of Experimental and Clinical Medicine, University “Magna Græcia” of Catanzaro, Viale Europa, 88100 Catanzaro, Italy; 2 Interdepartmental Center Veterinary Service for Human and Animal Health, CISVetSUA, University “Magna Græcia” of Catanzaro, 88100 Catanzaro, Italy; fabiocastagna@unicz.it (F.C.); musella@unicz.it (V.M.); britti@unicz.it (D.B.); 3 Department of Chemical, Biological, Pharmaceutical and Environmental Sciences, University of Messina, 98166 Messina, Italy; natalizia.miceli@unime.it (N.M.); mariafernanda.taviano@unime.it (M.F.T.); 4 Department of Health Sciences, University “Magna Græcia” of Catanzaro, Viale Europa, 88100 Catanzaro, Italy; 5 Department of Veterinary Medicine and Animal Productions, University of Naples Federico II, 80137 Napoli, Italy; boscoant@tiscali.it (A.B.); lrinaldi@unina.it (L.R.); 6 Centro Regionale per il Monitoraggio delle Parassitosi (CREMOPAR), Regione Campania, 84025 Eboli, Italy

**Keywords:** plant extracts, *Isatis tinctoria*, anthelmintic activity, egg hatch test, sheep, animal health, gastrointestinal nematodes

## Abstract

Sheep gastrointestinal nematode (GIN) infestation represents a limiting factor for sheep farming and milk production in Italy. The development of anthelmintic resistance to conventionally used drugs suggests the path towards the use of natural remedies as a possible alternative. The purpose of this study is to evaluate the in vitro anthelmintic efficacy of the hydroalcoholic extracts of basal leaves (It-BL), cauline leaves (It-CL) and flowers (It-F) of *Isatis tinctoria* (Brassicaceae), a spontaneous Sicilian species renowned as an important source of bioactive compounds. The dry extracts of the different parts of the plant were tested using the egg hatch test (EHT) in vitro to verify the efficacy against ovine GIN at different concentrations (1.00, 0.5, 0.25, 0.125 mg/mL). Thiabendazole and deionized water were used as positive and negative controls, respectively. The results obtained from EHT indicated that all the *I. tinctoria* extracts were highly effective (*p* < 0.0001) in inhibiting egg hatching within 48 h of exposure. The in vitro inhibitory effect was never less than 84% in all doses tested, and it was only slightly lower than the standard drug thiabendazole (95.6%). The current study documents the anthelmintic activity of *I. tinctoria* against sheep’s GIN, suggesting its application as alternative natural method to limit the use of antiparasitic drugs.

## 1. Introduction

Parasitic diseases, such as those caused by helminths, represent one of the main issues in livestock farms around the world. In particular, gastrointestinal nematodes (GIN) are constantly present in sheep and goat farms in Southern Italy [1]. Helminthiasis in livestock negatively affects productivity (i.e., reduced growth rate, weight loss and reduced fertility) and causes an associated economic damage that sums up with the veterinary costs and pharmacological treatments [2].

The therapies carried out with common anthelmintic drugs produced various problems such as compliance with withdrawal times, environmental pollution, and the progressive resistance that parasites are developing towards the most common chemical antiparasitic drugs.

Antiparasitic resistance appears to be more prevalent in sheep and goats and is present, but less prevalent, in horses and cattle [3]. Resistance to common antiparasitic drugs is a widespread problem all over the world, but it has different prevalence due to different uses and habits [3]. The resistance, more widespread in the most industrialized countries [4], is also recently becoming a problem in Italy and, even if rarely, in Southern Italy, where some concrete actions appear to have been effective in slowing the development of anthelmintic resistance (AR) [5]. The monitoring of GIN infection in sheep and other animals through regular diagnoses allows the use of targeted and alternative treatments in the early stages to block the occurrence of AR also in these anthelmintic resistance-free areas. This problem increases the need to find ecologically and economically sustainable solutions that provide for a non-chemical control of parasites. For this purpose, it may be useful to resort to the use of plants that grow spontaneously in the same geographical area in which animals are reared. The use of plants for the treatment of various diseases in humans and animals is historically linked to traditional medicine in different parts of the world [6,7] In addition, there are several plants that are used as anti-parasitic agents due to their properties in ethnoveterinary medicine [8,9]. Among the plants of which various beneficial properties have been demonstrated and which grow spontaneously in Sicily, there are several species belonging to the Brassicaceae family [10,11,12,13].

Brassicaceae have been the subject of numerous phytochemical investigations and studies on therapeutic potential against human and animal diseases [10,13,14,15,16]. *Isatis tinctoria* L., included in this family, has an ancient and well-documented history as an indigo dye, medicinal and an animal feeding plant [17].

For its properties this species, widely utilized for medical purposes in the Traditional Chinese Medicine [18,19,20] and recognized as a pharmacopeial plant in Europe [21], is of great interest, and this has led to an increase in its use in the European agricultural system [22,23].

With regard to the Brassicaceae that grow spontaneously in Sicily, and in particular with regard to *I. tinctoria*, previous in vitro studies have been carried out that described the composition and the cytotoxic and antioxidant properties [17,24,25].

The present work aimed to investigate the anthelminthic properties of the hydroalcoholic extracts obtained from the different plant parts of *I. tinctoria* growing wild in Sicily. Basal leaves, cauline leaves and the flowers extract were separately analyzed for their anthelmintic efficacy.

## 2. Materials and Methods

### 2.1. Plant Material and Extraction Procedure

**Figure 1:**
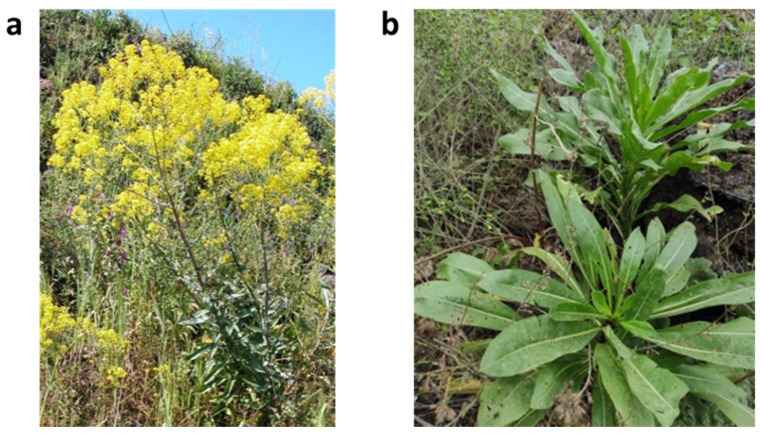
(**a**) *Isatis tinctoria* plant photographed in April showing flowers and cauline leaves. (**b**) Basal leaves are oblong-lanceolate, entire to toothed, and long-petioled.

The plant material was collected from *Isatis tinctoria* L., a Sicilian autochthonous plant, growing spontaneously in the Acireale geographical area (Catania, Sicily, Italy). Basal leaves (It-BL) were picked in November, cauline leaves (It-CL) and flowers (It-F) were harvested in April. In panel Figure 1a, it is possible to see the entire plant in April along with flowers and cauline leaves. Basal leaves (November) are visible in Figure 1b. Cauline leaves are narrower than basal leaves, gradually thinner upwards and simple, entire, sagittate, usually amplexicaul, with acute auricles. The flowers are gathered in a racemose inflorescence, with yellow petals, tetradynamous androecium, consisting of six stamens with two filaments shorter than the others.

The taxonomic identification of the plant materials was confirmed by Prof. Salvatore Ragusa, Department of Health Sciences, University “Magna Graecia” of Catanzaro (Italy). Voucher specimens are deposited in the Herbarium of the same Department, under accession number no. 327/11.

Briefly, freshly harvested leaves and flowers were immediately frozen; then, after the lyophilization, the plant material was sequentially extracted at 50 °C with dichloromethane (three times) and 70% methanol (three times), as described previously [26].

The filtrates of each hydroalcoholic extract were combined and evaporated to dryness by rotavapor. The yields of the dry extracts, referred to 100 g of lyophilized plant material, were 28.42%, 34.78% and 43.11% for It-BL, It-CL and It-F, respectively.

### 2.2. Parasitological Study

#### 2.2.1. Recovery of GIN Eggs

The fecal samples, necessary for the recovery of the GIN eggs, were taken directly from the rectal ampoule of naturally infected sheep in a farm located in Southern Italy and processed within 2 h. The technique used to isolate the eggs was that described by Coles et al. [27] and Bosco et al. [5].

In order to isolate the eggs from the feces, the fecal samples were first homogenized and then filtered by washing them under running water through 125, 63 and 38 µm mesh sieves. The GIN eggs retained on the last sieve were washed and centrifuged for 3 min at 170× *g* with distilled water. The supernatant obtained from the centrifugation was discarded.

Subsequently, a 40% sugar solution was added, and centrifugation was performed to bring out the eggs. The collected eggs were isolated in new tubes, mixed with distilled water, and then centrifuged several times to remove the pellets and obtain an aqueous solution containing the GIN eggs.

The eggs were examined under a microscope to see if the embryo formation process had not started. Ten aliquots of 0.1 mL containing 150 eggs each were prepared from the suspension obtained.

#### 2.2.2. Coprocultures

The genera of nematodes were identified using the morphological keys proposed by van Wyk and Mayhew [28] on larvae developed at the third stage (L3) in larval cultures, performed following the protocol of the Ministry of Agriculture, Fisheries and Food [29]. For the identification of the genus and for the estimation of the percentage of larvae present, 100 larvae in L3 present in the sample were evaluated; when the sample presented ≥ 100 larvae in L3, the percentage was calculated on the total number of L3. Each genus of nematode present in the sample was indicated as a percentage.

#### 2.2.3. Anthelmintic Efficacy

In order to estimate the in vitro anthelmintic efficacy of *I. tinctoria* dry extracts, the egg hatching test (EHT) has been used performing the procedure recommended by the World Association for the Advancement of Veterinary Parasitology (WAAVP) [27].

Each dry extract (It-CL, It-BL and It-F) was analyzed by making three replicates each and tested at decreasing concentrations. The results were compared with thiabendazole (TBZ, Sigma, Saint Louis, MO, USA) (0.5 and 0.25 mg/mL) and deionized water, used as positive and negative controls, respectively.

Neutral pH deionized water was used to prepare the thiabendazole solution following the protocol described by Von Samson-Himmelstjerna et al. [30].

The final concentrations in the EHT were prepared by adding 10 µL of each TBZ solution into 1.99 mL of a suspension with approximately 150 eggs/mL in water. The final TBZ concentrations used in this study were 0.2 and 0.5 μg/mL.

Each *I. tinctoria* dry extract was diluted in deionized water and assayed at different final concentrations obtained by two-fold dilutions (1.00, 0.5, 0.25, 0.125 mg/mL).

The 24-well tissue culture test plates (Corning Incorporated, Life sciences, Salt Lake City, USA) were incubated for 48 h at 25 °C. The incubation was then terminated by adding 10 µL of Lugol’s iodine solution to each well. The number of eggs hatched, embryonated and unembrionated were manually counted using a microscope (Leica, Wetzlar, Germany, ×20). The egg hatching (EHT) was estimated using the following formula described by Coles et al. [27]:

EHT = [(number of eggs)/(number of larvae + number of eggs)] × 100(1)

The statistical analysis was carried out with Microsoft excel and jmpSAS. Excel was used to apply student’s t test to evaluate the *p*-values. jmpSAS was used to create the graphs together with the error bars representation.

## 3. Results

The hydroalcoholic dry extracts obtained from basal leaves (It-BL), cauline leaves (It-CL) and flowers (It-F) of *I. tinctoria* were analyzed in vitro for their anthelmintic properties. It-BL, It-CL and It-F extracts were tested in three different and independent trials using, as positive control, the anthelmintic drug thiabendazole and, as negative control, deionized water.

The nematodes species composition in the fecal samples before the in vitro experiments was as follows: *Trichostrongylus* spp. (43%), *Haemonchus contortus* (23%), *Teladorsagia* spp. (21%), *Chabertia ovina* (7%) and *Cooperia* spp. (6%).

**Figure 2:**
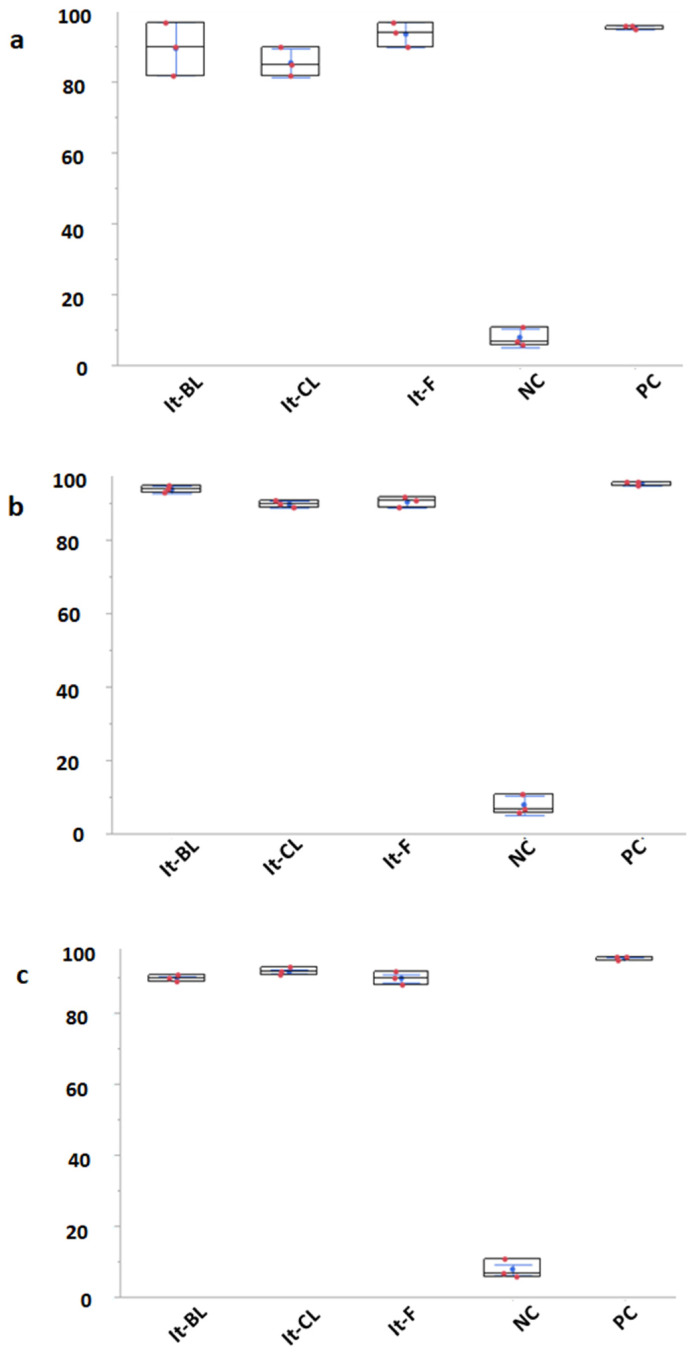

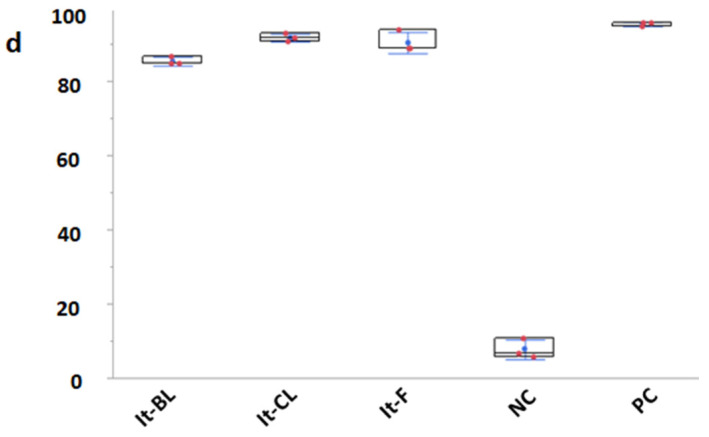
Effect of *I. tinctoria* hydroalcoholic dry extracts (It-BL, It-CL and It-F) and negative (NC) and positive (PC) controls against the growth of GINs at the following concentrations: (**a**) 1 mg/mL; (**b**) 0.5 mg/mL; (**c**) 0.25 mg/mL; and (**d**) 0.125 mg/mL. The *y*-axis represents the percentage of effectiveness.

Figure 2 clearly shows the consistent reduction in larvae growth caused by the It-CL, It-BL and It-F dry extracts in comparison with the negative control group (NC). When the extracts were tested at the concentration of 1 mg/mL, it was recorded an inhibition of larvae growth equal to 95% for the positive control and of 89% for It-BL, 85% for It-CL and of 93% for It-F. When the extracts were diluted 1 to 1 to obtain the 0.5 mg/mL solution, the reduction in larvae development was recorded as 94% for It-BL, 90% for It-CL and 91% for It-F. When the concentration was reduced to 0.25 and 0.125 mg/mL, the inhibition of larvae growth persisted at levels close or above 90%. It was recorded equal to 84% only in case of It-BL extract used at a concentration of 0.125 mg/mL. Comparing the efficacy of each extract at all the mentioned concentrations against the NC group, the statistical test always yields *p*-values below 0.0001. All the aforementioned results are resumed in Figure 2. The performed measurements clearly demonstrate the efficacy of all the tested *I. tinctoria* extracts in consistently reducing the ewes’ helminths larvae growth. The positive control thiabendazole showed a growth inhibition percentage of 95%, which represents only 5% more than the efficacy of the tested extracts.

## 4. Discussion

In this work, the hydroalcoholic dry extracts of *I. tinctoria* basal leaves (It-BL) cauline leaves (It-CL) and flowers (It-F) were investigated as natural alternatives against ewe’s gastrointestinal nematodes (GIN).

In a preliminary screening phase, in vitro assays are to be preferred because of their cost effectiveness and rapidity. These tests can be employed during the different parasitic life cycle stages to evaluate the different extracts’ anti-parasitic properties.

In our study, it was observed that in the eggs exposed to It-BL, It-CL and It-F extracts for 48 h, the embryonic development was present, but the eggs did not hatch. This clearly demonstrates the anthelmintic potential of *I. tinctoria* extracts against GIN isolated from fecal samples of naturally infected sheep. As visible in the results section (Figure 2), each plant part was able to inhibit, to levels at least as high as 84%, the egg hatching.

Our results agree with those described in a previous work, which reported the antiparasitic efficacy of *I. tinctoria* extracts against *Teledorsagia circumscripta*. In this study, Esteban-Ballesteros et al. demonstrated that the methanolic and aqueous extracts from *I. tinctoria* aerial parts inhibited egg hatching; however, the authors observed a strong activity of the extracts at much higher concentrations than those used in the present study [31].

The composition of each hydroalcoholic extract used in this study (It-BL, It-CL, and It-F) was previously analyzed via HPLC-PDA/ESI-MS [26]. The study highlighted that flavonoids represent the major class of the phenolic fraction. These molecules are mainly derivatives of flavones and of flavonols. The most abundant flavonoid in It-CL was vicenin-2, while luteolin-glucuronide and stellarin-2 were found to be the main compounds in the It-BL and It-F extracts, respectively. Moreover, the analysis led to the identification of four phenolic acids such as ferulic, sinapic, neo-chlorogenic and caffeic acid, being the latter the most abundant in all extracts [26].

Recent studies demonstrated that phenolic acids showed potent anthelmintic activity against both egg hatching and larval development of *H. contortus*. Among the tested compounds, caffeic acid and ferulic acid displayed the highest anthelmintic effects [32,33].

The eggs of gastrointestinal nematodes consist of three layers: the outer and middle layers are composed of protein fibrils and chitin, respectively, and a semipermeable inner layer is composed of lipids and glycoproteins [34]. Some authors suggest that polyphenolic compounds, such as condensed tannins and flavonoids, can bind to egg membrane proteins that are vital for the development and biological functions of the larvae. The proposed phenolic-protein interaction could be the cause of structural changes in the membrane, affecting its permeability, oxygen exchange and the release of substances and enzymes. This enhanced permeability may cause the degradation of the egg wall contrasting the proteins integrity and, as a consequence, the release of the larvae [35,36].

Based on above statements, it could be hypothesized that the strong activity of It-BL, It-CL and It-F can be attributed to the phenolic compounds, both flavonoids and phenolic acids, contained in the extracts; in particular, phenolic acids such as caffeic and ferulic acids could play a key role in the observed effects.

On the other hand, it has to be taken in consideration that several bibliographic data report that *I. tinctoria* exhibits a rich chemical profile characterized by a wide variety of bioactive components. In addition to phenolic compounds, numerous other molecules belonging to different chemical classes have been identified in this species such as alkaloids, polysaccharides, glucosinolates, carotenoids, volatile constituents, and fatty acids [17]. Thus, the involvement of other polar compounds contained in the It-BL, It-CL and It-F hydroalcoholic extracts cannot be ruled out.

In vitro studies should be supported by in vivo studies to evaluated bioavailability and any eventual toxic response. From the toxicity assay point of view, the extracts of *I. tinctoria* utilized in this study have been previously analyzed through the *Artemia salina* lethality bioassay, which represents a convenient system for the preliminary assessment of toxicity of the plant extracts. The results of the bioassay showed that none of the extracts displayed any toxicity against brine shrimp larvae, which indicated their potential safety [26].

According to the authors knowledge, to the date, no in vivo studies with this plant extract have been performed to evaluate its anthelmintic efficacy. The results here provided suggest that both *I. tinctoria* leaves and flowers may be suitable for nutraceutical and therapeutic applications as adjuvants in anthelmintic treatment in food-producing animals. In the authors’ opinion, *I. tinctoria* could be included in forage preparations to exploit its efficacy in helminthiasis control.

## 5. Conclusions

The results of the present study show that the hydroalcoholic extracts from basal leaves, cauline leaves and flowers of *I. tinctoria* interrupted the life cycle of GINs by inhibiting the egg hatching process and preventing the development of infecting larvae.

These in vitro results should be confirmed in vivo considering the possibility of using the hydroalcoholic extracts or entire parts of *I. tinctoria* as feed or food additives in infected sheep for GIN control.

## Data Availability

No extra supporting data are needed for this manuscript.
